# Reframing the Rights to Care through the Ethics of Care

**DOI:** 10.1093/geroni/igaf122.4001

**Published:** 2025-12-31

**Authors:** Aeji Jang

**Affiliations:** Dongguk University, Gyeongju-si, Kyongsang-bukto, Republic of Korea

## Abstract

The purpose of this study is to clarify the rights to care for older people and caregivers from the perspective of the public ethics of care. Despite increased attention and efforts toward caring for older people in welfare states, concerns about their care persist. By assuming the ideal of independent citizens, welfare states have overlooked the fact that everyone experiences dependency and lives within caring relationships. Furthermore, attempts to manage care within the private sphere in a cost-effective way have led to neglecting the intrinsic value of care work. This suggests the need for a fundamental shift in our approach to designing care policies. As public ethics of care does not exclude individuals who live with unavoidable dependency, such as older adults, it offers a normative framework that considers care as a right. It distinguishes between the unavoidable dependency of care receivers and the derived dependency of caregivers, emphasizing the state’s responsibility to support both. For conceptualizing ‘the rights to care’, we conducted a narrative literature review. An in-depth review of studies and books about public ethics of care was carried out, with a focus on the lived realities of care-receiving and caregiving. Finally, this study distinguishes the rights to care as ‘rights to receive’ for older people and ‘rights to provide’ for caregivers, with four sub-principles: Access, Individuality, Compensation, and Security. This study not only contributes to the development of care ethics but also provides a practical and policy foundation for an aging society.

